# Bringing dead proteins back to life

**DOI:** 10.7554/eLife.02189

**Published:** 2014-02-11

**Authors:** Brandon A Wustman, John W Steele, Eric R Sjoberg, Anthony C Stevens

**Affiliations:** 1 **Brandon A Wustman** is at OrPhi Therapeutics, Carlsbad, United Statesbwustman@orphitherapeutics.com; 2 **John W Steele** is at OrPhi Therapeutics, Carlsbad, United States; 3 **Eric R Sjoberg** is at OrPhi Therapeutics, Carlsbad, United States; 4 **Anthony C Stevens** is at OrPhi Therapeutics, Carlsbad, United States

**Keywords:** myosin, protein folding, allostery, pharmacological chaperone, Dictyostelium, Human, rat

## Abstract

A small molecule called EMD 57033 can repair motor proteins that have stopped working as a result of stress.

**Related research article** Radke MB, Taft MH, Stapel B, Hilfiker-Kleiner D, Preller M, Manstein DJ. 2014. Small molecule-mediated refolding and activation of myosin motor function. *eLife* **3**:e01603. doi: 10.7554/eLife.01603

**Image** Motor proteins walk along filaments made from actin (labelled in green) to contract muscle cells

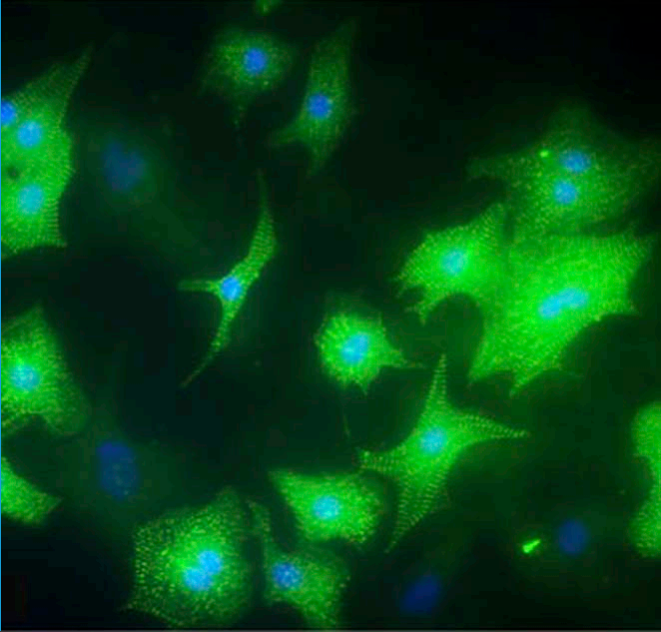

The muscles in our bodies, including those that make up our hearts, contain large numbers of ‘motor proteins’ called myosins. Motor proteins generate forces inside cells by ‘walking’ along filaments of a protein called actin, and these forces are used to contract muscles, to traffic cargo within the cells or to segregate chromosomes during cell division. These activities subject the myosin proteins to physical stress that, together with the extremes of pH and temperature found in the cell, can lead to the motor proteins losing their shape. Misfolding can also result from genetic mutations, with extremely serious consequences for health: about one-third of cases of familial hypertrophic cardiomyopathy—a genetically inherited heart disease that affects 1 in 500 people worldwide (Maron et al., 1995; Maron, 2002)—are caused by mutations in a component of cardiac β-myosin. Many of these mutations are thought to impair the ability of this motor protein to bind to actin or release the energy supplied by ATP.

Now in *eLife,* Dietmar Manstein and colleagues at the Hannover Medical School and DESY Research Centre—including Michael Radke and Manuel Taft as joint first authors—report that a small molecule called EMD 57033, which can improve the performance of myosin, can even restore the activity to ‘dead’ myosin protein that has been rendered inactive by stress-induced misfolding (Radke et al., 2014).

Compounds that activate myosin and have the ability to promote muscle contractions are said to display a positive inotropic effect: examples include EMD 57033 and Omecamtiv Mecarbil (which is currently undergoing phase II clinical trials for the treatment of heart failure). These compounds have been used to study how myosins generate a pulling force. Further, it has been previously reported that the inotropic effects of EMD 57033 were the result of increases in the sensitivity of myosin to calcium ions (which enter into muscle cells when they need to contract; Solaro et al., 1993). However, the Hannover group provide evidence for a different and novel mechanism of action.

Through a series of in vitro experiments, Radke, Taft et al. show that EMD 57033 likely binds to the same region of myosin as Omecamtiv Mecarbil. They also show that the presence of EMD 57033 increases the rate of ATP binding, and that the resulting increase in energy release leads to higher force production. The binding of EMD 57033 to myosin also increases its ability to withstand high temperatures and, remarkably, mediates the refolding of at least some stress-induced misfolded proteins—akin to bringing these proteins ‘back from the dead’ (Figure 1). These stabilizing and refolding properties could help increase the number of functional myosin motors, while also reducing the formation of insoluble aggregates of myosin (which can be toxic to cells).

**Figure 1.**
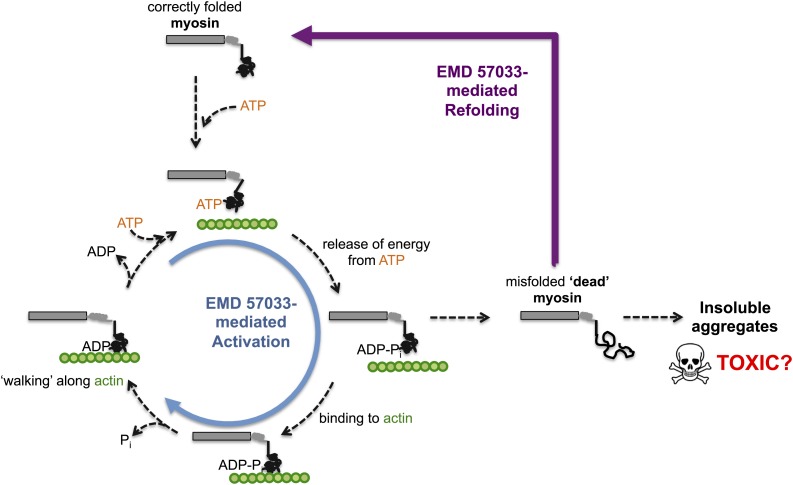
The small molecule EMD 57033 mediates both activation and refolding of the myosin motor protein. A myosin motor protein binds to a molecule of ATP, and releases the energy it contains by splitting it into an ADP molecule and a phosphate ion (Pi). After binding to a filament of actin (shown in green), the motor protein then uses this energy to ‘walk’ along the filament. Repeating this cycle generates a pulling force that can cause a muscle cell to contract—however, this effort, as well as other stresses within the muscle cell, can promote misfolding and a loss of motor function. The accumulation of misfolded ‘dead’ myosin can lead to the formation of protein aggregates, which can have potentially toxic effects. Radke, Taft et al. show that when EMD 57033 (not shown) binds to cardiac β-myosin it increases the rate of ATP binding and energy release (blue circular arrow), which accelerates the motor proteins progression along the actin filament. Remarkably, EMD 57033 can also refold some forms of misfolded myosin back to a properly folded and functional state (purple arrow)—an activity that has not been observed before for similar small molecules.

To maintain the levels of different proteins within ranges that allow the cell to function correctly, the synthesis, maintenance and breakdown of a wide variety of proteins need to remain in balance. However, cellular stresses—such as increased temperature and mutations that reduce protein stability—can reduce the efficiency of protein folding and refolding, resulting in the accumulation of unfolded or misfolded proteins. These unfolded proteins are more likely to form aggregates, which will impair the ability of cells to breakdown proteins.

The effects of EMD 57033 on myosin are similar to the effects typically associated with small molecules called pharmacological chaperones that selectively bind and stabilize mutant proteins. These molecules represent a new class of treatment for patients with diseases that result from protein destabilization, unfolding or misfolding. However, EMD 57033 is the first example of a pharmacological chaperone that is able to convert stress-induced misfolded protein back into a fully functional form.

The work of the Hannover team is significant as it reveals a new pharmacological chaperone-like mechanism of action that may, at least in part, explain the inotropic effect of EMD 57033. The team’s findings also represent the first demonstration of a pharmacological chaperone-induced protein refolding with subsequent restoration of the protein’s function. A better understanding of EMD 57033 could lead to additional studies in at least three areas: the study of force generation mechanisms; the development of improved biosensors; and the interpretation of the clinical effects observed in trials of other myosin activators (such as Omecamtiv Mecarbil). A better understanding of if and how EMD 57033 can improve the function of mutant forms of myosin might also lead to improvements in the design of clinical trials by identifying those patients who are predicted to benefit the most from treatment with a specific inotropic agent.
